# A Single Dose of Azithromycin and Inflammation in Infants: A Cluster-Randomized Placebo-Controlled Trial

**DOI:** 10.4269/ajtmh.26-0486

**Published:** 2026-11-10

**Authors:** Rikhard Ihamuotila, Fadima Haidara, Oumar Samaké, Juho Luoma, Yin Bun Cheung, Laura Adubra, Dagmar Alber, Awa Traoré, Jane Juma, Camilla Ducker, Ulla Ashorn, Per Ashorn, Samba Sow, Yue-Mei Fan

**Affiliations:** ^1^ Center for Child, Adolescent and Maternal Health Research, Faculty of Medicine and Health Technology, Tampere University and Tampere University Hospital, Tampere, Finland;; ^2^ Center for Vaccine Development–Mali, Bamako, Mali;; ^3^ Great Ormond Street Institute of Child Health, University College London, London, United Kingdom;; ^4^ Tro Da Ltd, Caerphilly, United Kingdom

## Abstract

Azithromycin mass drug administration (MDA) reduces under-5 mortality in some African settings. The biomechanisms behind the mortality reduction are unknown. The aim of this study was to test the hypothesis that a dose of azithromycin reduces inflammation in infants in a West African setting. This cluster-randomized, double-blind, placebo-controlled trial was part of a large-scale trial in Mali conducted to test the effect of azithromycin MDA on infant mortality (LAKANA trial, ClinicalTrials.gov NCT04424511). In the LAKANA trial, villages were randomly assigned to placebo or azithromycin groups. All participants, investigators, and study personnel were masked. Whole-blood C-reactive protein (CRP) concentration was measured in 4- to 11-month-old infants in a subset of villages right before and 2 weeks after receiving either placebo or azithromycin. CRP values were log-transformed, and mixed-effects regression with random intercepts for clusters and identity link was used to compare the groups. Among 1,615 infants in 72 villages, the baseline geometric mean CRP level was 1.76 mg/L in the placebo group (*n* = 696) and 1.81 mg/L in the azithromycin group (*n* = 919). On day 14, the mean CRP level was 1.67 mg/L in the placebo group and 1.47 mg/L in the azithromycin group, accounting for an average 14% lower CRP with azithromycin (95% CI: 1%–26%; *P* = 0.048). There was no statistically significant difference in prevalence of elevated CRP levels (>5 mg/L). Those with two or more morbidities seemed to benefit most, with 42% lower CRP levels on average with azithromycin treatment (95% CI: 1%–67%; *P* = 0.047). These findings support the hypothesis that a single dose of azithromycin reduces inflammation in Malian infants.

## INTRODUCTION

Nearly 5 million children under 5 years old die yearly, with more than half of these deaths occurring in sub-Saharan Africa.^1^ In this region, mass drug administration (MDA) of azithromycin is a promising intervention to reduce child mortality.^2^^–^^5^ Because of the risk of antimicrobial resistance, understanding the mechanism behind mortality reduction with azithromycin MDA has been identified as a key priority.^6^

As a broad-spectrum antibiotic with excellent tissue penetration and a long half-life, azithromycin can potentially be used to treat, eradicate, or reduce the pathogen burden of many bacteria.^7^ Additionally, azithromycin has some antiviral properties against rhinoviruses, influenza, coronaviruses, and enteroviruses, among others.^8^ The weak antimalarial effect of azithromycin is also well established.^9^ Interestingly, azithromycin has a vast array of nonantibiotic anti-inflammatory properties related to macrophages, neutrophils, various cytokines, and epithelial integrity.^8^^,^^10^

In the context of azithromycin MDA for child survival, some trials have observed reductions in malaria, pneumonia, diarrhea, general clinic visits, and antibiotic prescribing.^11^^–^^15^ These often marginal morbidity and mortality reductions do not entirely explain why a rarely given single dose of azithromycin reduces mortality for a prolonged time.^16^ Evidence of a spillover effect on survival among untreated children in azithromycin MDA villages suggests a possible herd protective effect. However, a comprehensive biomechanistic explanation for azithromycin MDA effects has yet to be discerned.^17^

Inflammation markers could serve as proxies for acute or chronic pathogenic or immunologic burden in individuals receiving azithromycin MDA. This study aimed to test the hypothesis that a single dose of azithromycin reduces inflammation among infants in a low-income West African setting.

## MATERIALS AND METHODS

### Trial design and oversight.

This substudy was nested within the LAKANA trial (ClinicalTrials.gov NCT04424511, registered June 11, 2020), a large-scale placebo-controlled, cluster-randomized trial conducted in Western Mali to test the effect of azithromycin MDA on infant mortality. The trial protocol, statistical analysis plan, and standard operating procedures have been prospectively published.^18^^–^^20^

In the LAKANA trial, villages (clusters) were randomly assigned to three arms: placebo, azithromycin MDA two times yearly, and azithromycin MDA four times yearly. The present substudy was conducted during the fourth MDA round in the included substudy villages (i.e., 9 months after the first MDA and 3 months after the previous MDA in those villages). The same study procedures were implemented at two sites in Western Mali to capture variations across years, seasons, and areas: first in the Kita Region between February 17 and May 30, 2022 and then repeated in the Koulikoro Region between July 24 and August 29, 2023. For the present substudy, all villages receiving azithromycin during the fourth MDA were combined into the azithromycin group, and those receiving placebo during the MDA were combined into the control group.

### Randomization and blinding.

Villages were randomly assigned to control, twice-yearly azithromycin, or quarterly azithromycin arms in a 3:4:2 ratio, respectively. Randomization was performed at a public allocation event, where village representatives drew lottery tickets with letter codes. The letter code defined the trial arm of a village. All investigators, participants, caregivers, village leaders, study site and laboratory personnel were masked. Only the external partners (the chair of the trial data safety and monitoring board, key Pfizer personnel, and an external statistician) facilitating randomization were unmasked. To maintain blinding, the placebo and azithromycin substances had similar packaging and were indistinguishable from each other. Details of the randomization and masking have been published in the main trial.^21^

### Eligibility.

The secondary outcomes sample of the LAKANA trial included 59 villages in the Kita Region and 14 Kati District villages in the Koulikoro Region in Western Mali, chosen for their proximity to the Kita and Bamako study headquarters, which made timely transportation and storage of biological samples possible. As part of the main trial, infants aged 1 to 11 months with no known macrolide allergy, weighing at least 3.0 kg, and with no severe illness were eligible to receive the study drug. For the present substudy, infants aged 4 to 11 months (120–364 days) were eligible for biological sample collection. Severely ill infants and those with C-reactive protein (CRP) levels greater than or equal to 50 mg/L or hemoglobin (Hb) levels less than 8.0 g/dL were referred to the nearest health facility for further diagnostics and management. Eligible infants referred because of high CRP or low Hb levels received the study drug and were included in the analysis regardless of whether they visited the nearest health facility or received additional treatment.

### Intervention.

Data collectors visited all the households in the substudy villages as part of the main LAKANA trial and identified all treatment-eligible infants. Those aged 4 to 11 months and their caregivers were accompanied to a temporary village facility, where a study nurse first took blood samples. Infants were then weighed with an electronic scale (ADE Model M111600-01; ADE Germany GmbH; Hamburg, Germany) and given reconstituted oral suspension of the study drug (either azithromycin at a dosage of 20 mg/kg or placebo).

### Sample collection and CRP measurement.

A study nurse performed a heel prick with a small lancet and obtained 10 *µ*L of capillary blood. The capillary blood was transferred into a reagent cuvette and then inserted into the rapid test instrument (QuickRead go® wrCRP+Hb; Aidian; Espoo, Finland). The instrument automatically performed the optical measurement and provided CRP and Hb results within minutes. Quality control procedures were conducted according to manufacturer recommendations to ensure accuracy and reliability of the measurements. The study nurse documented the test results twice in the electronic data collection system.

After 14 days, data collectors returned to the households, identified the infants assessed at the baseline visit, and accompanied the infants and their caregivers to the temporary village facility. Blood CRP levels were measured again with the rapid test machine. On this second visit, caregivers were also interviewed about any adverse events related to study drug administration, which have been reported previously.^21^

### Outcomes and effect modifiers.

The main outcomes included whole-blood CRP concentration (mg/L) and prevalence of elevated CRP (>5 mg/L). Possible effect modifiers included child weight-for-age *z*-score, age, sex, place and timing (Kita in 2022 versus Kati in 2023), household asset index, and water, sanitation, and hygiene (WASH) index. Details of the WASH and asset indices are described in the trial statistical analysis plan.^19^

### Comorbidity.

Comorbidity was defined as having two or more morbidities. The determined morbidities included underweight (weight-for-age *z*-score <2), high environmental enteric (EE) score (8–10) as an indicator of more severe environmental enteric dysfunction (EED), severe anemia (Hb <7.0 g/dL), and malaria parasitemia (polymerase chain reaction [PCR] cycle threshold value below 38). These four morbidities were combined in 10 ways to form comorbidity combinations (e.g., malaria and severe anemia; malaria and underweight; malaria, underweight, and severe anemia). Comorbidity subgroup analyses were intended to be exploratory and hypothesis-generating.

To determine morbidities, stool samples for EED biomarkers and dried blood spot samples for malaria PCR were also obtained at the temporary village facility right before and 14 days after participants received the study drug. The sample collection procedures and laboratory analyses for EE score and malaria PCR are reported elsewhere.^22^^,^^23^

### Sample size.

The 59 Kita villages were estimated to have 333 to 450 infants aged 1 to 11 months in each study arm, and a total of 1,000 to 1,350 infants. This offered a statistical power of 80% to detect an effect size of 0.2 to 0.3 SD for the comparison of azithromycin versus placebo at a 5% two-sided type 1 error rate, assuming that the inter-cluster correlation was not larger than 0.03.^19^ The 14 Kati villages were added prospectively while the LAKANA study was already ongoing to further increase the sample size and catch any regional or seasonal effect. One Kita village served as a pilot village for substudy activities and was therefore excluded from analyses.

## STATISTICAL ANALYSES

A mixed-effects linear regression with random intercepts for villages was used, with baseline outcomes (CRP concentration or prevalence) and intervention group used as independent variables and day 14 outcomes used as dependent variables. Because of the skewness of CRP concentration distributions, CRP values were log-transformed for the regression analyses, and geometric means and geometric SDs were reported. Logistic regression was used for the prevalence of elevated CRP levels (binary outcome). Marginal RRs were predicted from the obtained model coefficients, and their CIs were obtained using the delta method.

For effect modifiers, the statistical significance of interactions was determined using a predefined *P*-for-interactions of less than 0.1. To demonstrate changes in the CRP value distribution with azithromycin, density plots of baseline and day 14 log-transformed CRP values were superimposed in the control and azithromycin groups separately. Statistical analyses were conducted in the R programming language 4.5.2 (R Foundation; Vienna, Austria).

## RESULTS

At baseline, a total of 1,615 eligible infants across 72 villages were treated (696 infants in the control group and 919 in the azithromycin group; Figure 1). CRP results were obtained for 93.3% of the treated infants. The geometric mean of the CRP levels was 1.76 mg/L in the control group and 1.81 mg/L in the azithromycin group (Table 1). The prevalence of elevated CRP levels (>5 mg/L) was common: 13.9% in the placebo group and 16.5% in the azithromycin group. The prevalence of underweight (weight-for-age *z*-score ≤2) was also common: 18.2% in the control group and 17.0% in the azithromycin group. The prevalence of any combination of two or more known morbidities was 9.4% in the 2022 Kita villages sample. The prevalence of specific comorbidity combinations was less than 5% in the control and azithromycin groups. Baseline mean CRP was similar between years and regions: 1.87 mg/L in 2022 in the Kita villages and 1.60 mg/L in 2023 in the Kati villages (not shown). Baseline mean CRP levels were also similar between the Kati placebo group and the Kati twice-yearly group (which received azithromycin in the previous MDA but placebo during the present substudy MDA): 1.63 mg/L and 1.57 mg/L, respectively.

**Figure 1.**
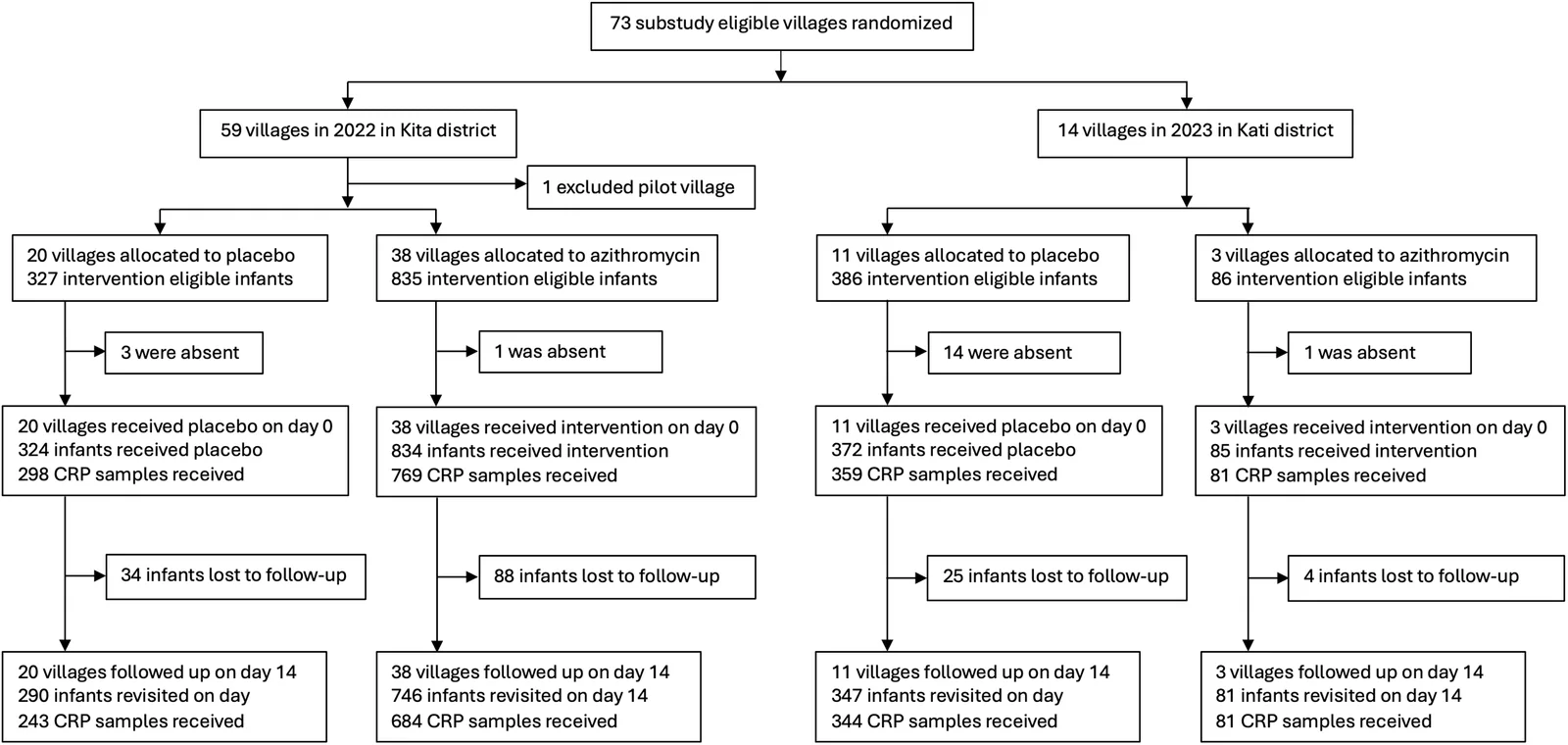
Participant flowchart.

**Table 1:** Baseline characteristics (day 0) of infants by treatment group

Variable	Control	Azithromycin
Number of infants	696	919
Average number of infants per village (SD)	22.5 (16.7)	22.4 (22.2)
Proportion of girls	50.0%	48.3%
Mean (SD) age (months)	7.9 (2.3)	7.7 (2.3)
Mean (geometric) CRP (GSD), mg/L	1.76 (2.57)	1.81 (2.83)
Mean (geometric) CRP (GSD) among those with CRP >5, mg/L	9.5 (1.84)	10.4 (1.93)
Mean (SD) WAZ	−1.02 (1.13)	−0.96 (1.14)
Prevalences, *n*/*N* (%)		
Prevalence of elevated CRP (>5 mg/L)	91/657 (13.9)	140/850 (16.5)
Prevalence of malaria*	27/288 (9.4)	74/750 (9.9)
Prevalence of severe anemia	72/657 (11.0)	78/850 (9.2)
Prevalence of underweight (WAZ ≤2)	127/696 (18.2)	156/918 (17.0)
Prevalence of high EE score*	49/274 (17.9)	140/683 (20.5)
Comorbidity combinations (only Kita area 2022 sample),* *n*/*N* (%)		
Proportion with both high EE score and underweight	10/274 (3.6)	21/682 (3.1)
Proportion with both high EE score and severe anemia	3/273 (1.1)	11/682 (1.6)
Proportion with both severe anemia and underweight	7/298 (2.3)	16/768 (2.1)
Proportion with both malaria and underweight	8/288 (2.8)	16/749 (2.1)
Proportion with both malaria and high EE score	6/264 (2.3)	20/669 (3.0)
Proportion with both malaria and severe anemia	5/286 (1.7)	8/748 (1.1)
Proportion with high EE score, malaria, and underweight	3/264 (1.1)	7/668 (1.0)
Proportion with high EE score, severe anemia, and underweight	2/273 (0.7)	3/681 (0.4)
Proportion with malaria, severe anemia, and underweight	2/286 (0.7)	3/747 (0.4)
Proportion with high EE score, severe anemia, malaria, and underweight	1/263 (0.4)	1/667 (0.1)

CRP = C-reactive protein; EE score = environmental enteric score; GSD = geometric SD; high EE score = EE score of 8 to 10; severe anemia = hemoglobin <7.0 g/dL; WAZ = weight-for-age *z*-score.

* Only the Kita 2022 sample that has EE score and malaria status available (1,158 infants in 58 villages).

At reassessment 14 days after treatment, a total of 1,464 infants were revisited (Figure 1). Loss to follow-up was 8.5% (*n* = 59) in the control group and 10.0% (*n* = 92) in the azithromycin group. On average, infants were revisited 15.1 days (SD: 2.8 days) after treatment.

### Primary outcomes.

On day 14, the mean CRP level was 1.67 mg/L in the control group and 1.47 mg/L in the azithromycin group, with a ratio of means estimate of 0.86 (95% CI: 0.74 to 0.99; *P* = 0.048; Table 2). The prevalence of elevated CRP (>5 mg/L) was 10.1% in the control group and 13.3% in the azithromycin group, with an RR of 1.17 (95% CI: 0.77 to 1.77; *P* = 0.46; Supplemental Table 1).

**Table 2:** Geometric mean (geometric SD) C-reactive protein (mg/L) at day 14 by treatment group and comorbidity combination

Variable	Control (GSD)	Azithromycin (GSD)	Ratio of Means Estimate (95% CI)*	*P*-Value
Mean CRP	1.67 (2.42)	1.47 (2.88)	0.86 (0.74 to 0.99)	0.048^†^
Mean CRP among infants with two comorbidities (only Kita 2022 sample, *n* = 1,158 infants)				
Any two or more morbidities (*n* = 87)	2.99 (2.68)	1.63 (3.00)	0.58 (0.33 to 0.99)	0.047^†^
Underweight and high EE score (*n* = 31)	2.74 (3.29)	1.55 (2.86)	0.52 (0.22 to 1.24)	0.14
Underweight and severe anemia (*n* = 23)	4.44 (2.70)	1.22 (2.70)	0.31 (0.11 to 0.86)	0.025^†^
Underweight and malaria (*n* = 24)	3.09 (3.00)	1.91 (3.12)	0.72 (0.28 to 1.82)	0.48
High EE score and severe anemia (*n* = 14)	3.33 (2.40)	1.36 (2.42)	0.53 (0.13 to 2.08)	0.36
High EE score and malaria group (*n* = 26)	1.84 (2.64)	1.77 (3.70)	1.11 (0.39 to 3.17)	0.85
Malaria and severe anemia (*n* = 13)	3.35 (3.66)	1.42 (2.71)	0.61 (0.17 to 2.15)	0.44
Mean CRP among infants with three comorbidities (only Kita 2022 sample)				
High EE score, malaria, and underweight (*n* = 10)	1.73 (3.74)	1.54 (3.78)	0.93 (0.23 to 3.86)	0.92
High EE score, severe anemia, and underweight (*n* = 5)	3.57 (3.41)	1.10 (1.10)	0.32 (0.05 to 2.08)	0.23
Malaria, severe anemia, and underweight (*n* = 5)	5.34 (6.02)	1.20 (N/A)	0.35 (0.03 to 4.35)	0.41
Mean CRP among infants with four comorbidities (only Kita 2022 sample)				
High EE score, severe anemia, malaria, and underweight (*n* = 2)	1.50 (N/A)	1.20 (N/A)	0.98 (0.05 to 17.87)	0.99

CRP = C-reactive protein; EE score = environmental enteric score; GSD = geometric SD; high EE score = EE score of 8 to 10; severe anemia = hemoglobin <7.0 g/L; underweight = weight-for-age *z*-score lower than 2. All means are geometric means.

* Ratio of geometric means estimate accounts for baseline values and is the regression coefficient calculated with a mixed-effects regression model with random intercepts for clusters.

^†^ *P*-value <0.05, not corrected for multiple comparisons.

Density plots of log-transformed CRP values were similar at baseline and on day 14 among controls, whereas a general shift in log-transformed CRP toward 0 is visible in the azithromycin group (Supplemental Figure 1). The density plot shift toward 0 with azithromycin is most prominent at values closer to 0. The prevalence of CRP levels greater than 1 mg/L on day 14 was significantly lower with azithromycin (RR: 0.81; *P* <0.001; Supplemental Table 1).

### Comorbidity analyses and effect modifiers.

Among the 87 infants with comorbidity (any two or more morbidities), the mean CRP level on day 14 was 2.99 mg/L in the control group and 1.63 mg/L in the azithromycin group, with a ratio of means of 0.58 (95% CI: 0.33 to 0.99; *P* = 0.047) favoring azithromycin (Table 2). Mean CRP concentration point estimates for comorbidity groups were consistently lower in children who had received azithromycin than in children who had received a placebo (except for infants with both high EE score and malaria). Of all the comorbidity combinations, CRP levels were significantly lower only in infants with any comorbidity and in infants with severe anemia and underweight.

All point estimates for the effect modifier subgroups favored azithromycin (Figure 2). The effect modifiers that met the predefined *P*-for-interactions <0.1 were place and timing (Kita in 2022 versus Kati in 2023) and comorbidity (*P*-for-interactions: ∼0.09 for both effect modifiers).

**Figure 2.**
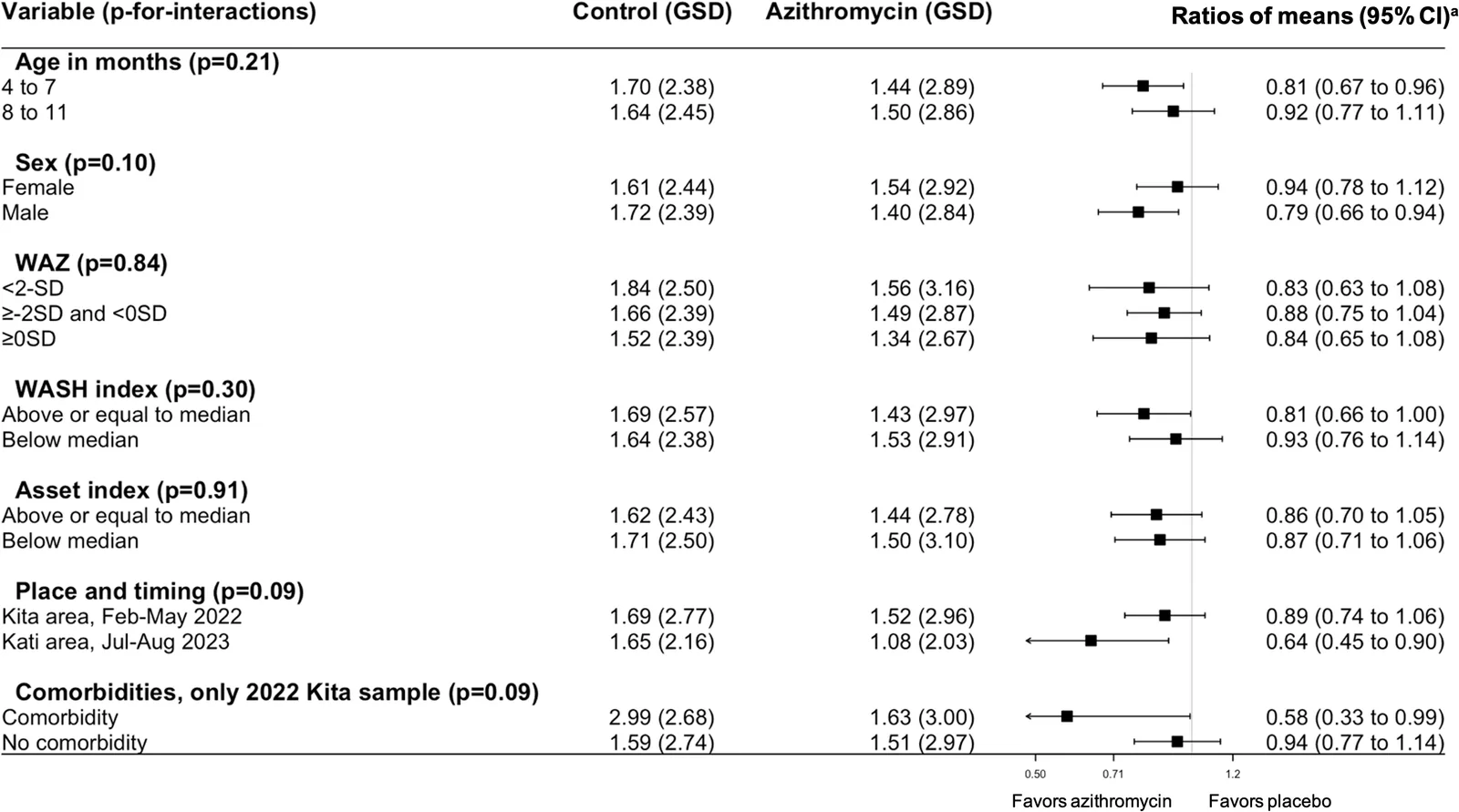
Geometric mean (GSD) CRP (mg/L) at day 14 for each effect modifier by treatment group. All means are geometric means. CRP = C-reactive protein; GSD = geometric SD; WASH = water, sanitation, and hygiene; WAZ = weight-for-age *z*-score. * Ratio of geometric means estimate accounts for baseline values and is the regression coefficient calculated with a mixed-effects regression model with random intercepts for clusters.

## DISCUSSION

The aim of this study was to test the hypothesis that a single dose of azithromycin reduces general inflammation among infants in a low-income West African setting. In a sample of 1615 infants across 72 villages, CRP concentrations were on average 14% lower with azithromycin treatment compared with placebo after 2 weeks. Children with two or more morbidities had 42% lower CRP concentrations on average with azithromycin treatment.

A weakness of this study is that a more acute (hours to a few days) reduction in CRP levels might have been missed because concentrations were measured only at 2 weeks, whereas the half-life of plasma CRP is ∼19 hours.^24^ However, reduced CRP levels at 2 weeks suggest a prolonged effect on inflammation. Some placebo group infants in the twice-yearly villages in the Kati District had received azithromycin within the last 6 months in the previous MDAs. This was nevertheless unlikely to bias the results because any sustained effect from previous MDAs should have resulted in a smaller effect on CRP, not the larger effect observed in Kati compared with Kita. Additionally, baseline CRP was similar between the twice-yearly and placebo groups in the Kati District, justifying the combining of the trial arms. The comorbidity analyses were exploratory; thus, even if ratios of means were smaller in many comorbidity subgroups than in the general sample, the findings should be regarded as hypothesis-generating rather than as actual effects. Additionally, comorbidity group sample sizes were small, resulting in wide CIs. The most remote LAKANA villages were not represented because the substudy villages were selected closer to the study laboratories. However, given the rural and relatively homogeneous trial setting, major differences between substudy and overall LAKANA villages were unlikely.

Others have observed reductions in CRP when testing the effects of azithromycin on cystic fibrosis, HIV-associated lung disease, influenza, chronic obstructive pulmonary disease, and even in coronary artery stenting and lung transplantation.^25^^–^^30^ Nonetheless, all these studies have tested the effects of azithromycin in the context of a specific disease or condition. One non-disease-specific study, in which sulphadoxine–pyrimethamine (SP) was compared with azithromycin plus SP for birth outcomes in pregnancy, observed a lower CRP concentration with azithromycin.^31^ Findings of this present study are consistent with these previous studies but extend the evidence on azithromycin and CRP in a non-selected, asymptomatic child population.

Azithromycin MDA targeting either child mortality or trachoma has yielded reductions in morbidity and deaths due to malaria, pneumonia, and diarrhea.^12^^–^^15^^,^^32^ Reductions in some specific pathogens, such as *Haemophilus influenzae* type b*, Bordetella pertussis*, and multiple enteropathogens have been observed after azithromycin MDA.^33^^–^^35^ Fewer clinic visits and antibiotic prescriptions, as well as a reduction in morbidity symptoms, have also been reported.^11^^,^^36^ The LAKANA EED substudy, from which the EE scores of the Kita 2022 sample are taken, also demonstrated a reduction in EED after azithromycin MDA.^23^ Based on these many studies reporting signs of reduced pathogenic load, it is not surprising that CRP or other inflammatory marker concentrations may be reduced with azithromycin MDA.

Azithromycin and other macrolides are known to affect the inflammation cascade through multiple pathways, which may explain the observed reduction in CRP concentration.^8^^,^^10^^,^^37^ Because both the production and effects of CRP are closely related to interleukins (IL), macrophages, neutrophils, lymphocytes, and tissue-level damage, a reduction in CRP levels due to azithromycin could occur in several ways.^38^ Production of CRP is primarily induced by IL-6 but also by the cytokines IL-1, IL-8, and tumor necrosis factor-α.^38^ Receiving azithromycin is associated with reduced concentrations of these four CRP-inducing cytokines in vivo, which could in itself explain a reduction in CRP concentration observed in the present study.^28^^,^^39^ Conversely, the reductions in specific pathogens and morbidity reported in other azithromycin MDA studies, together with the finding of this study that infants with two or more morbidities benefit the most, could indicate an effect on tissue-level burden of infectious load or inflammation.

It is possible that children with comorbidities may be the ones who benefit most from azithromycin MDA. There is some compelling evidence that mortality reduction with azithromycin MDA is enhanced in communities with higher mortality and morbidity.^40^^,^^41^ Supporting this notion is the almost halving of CRP concentrations among those with two or more morbidities in the study sample. Whether CRP reduction arises indirectly, through treatment of underlying infections and reduced tissue-level inflammation, or directly via immunomodulatory actions, such as inhibition of pro-inflammatory signaling pathways, remains difficult to distinguish.

This substudy was conducted among infants only, limiting generalizability to older children and to MDA programs targeting all children under 5 years old. A possible herd protection effect may also have been missed because only a small subset of the community was treated. Additional longitudinal azithromycin MDA studies are needed to explore specific inflammation parameters (such as cytokines and leukocyte types) in relation to specific pathogens and comorbidity. Such biomechanistic studies of azithromycin MDA should ideally be conducted as part of trials treating all children under 5 years old, with an eye on the proposed community spillover effect and antimicrobial resistance.^6^^,^^17^^,^^42^

## Supplemental Materials

10.4269/ajtmh.26-0486 Supplemental Materials
