# Supplementary material for: A Single Dose of Azithromycin and Inflammation in Infants: A Cluster-Randomized Placebo-Controlled Trial
Source: Am J Trop Med Hyg. 2026 Nov 10:tpmd260486. Online ahead of print. doi: 10.4269/ajtmh.26-0486 (PMC13770337; doi:10.4269/ajtmh.26-0486)
Supplement: Supplementary file 1 [file tpmd260486.SD1.pdf]

Supplementary

Supplemental Figure 1. Density plots of log-transformed CRP values by treatment group with baseline and day 14 distributions superimposed

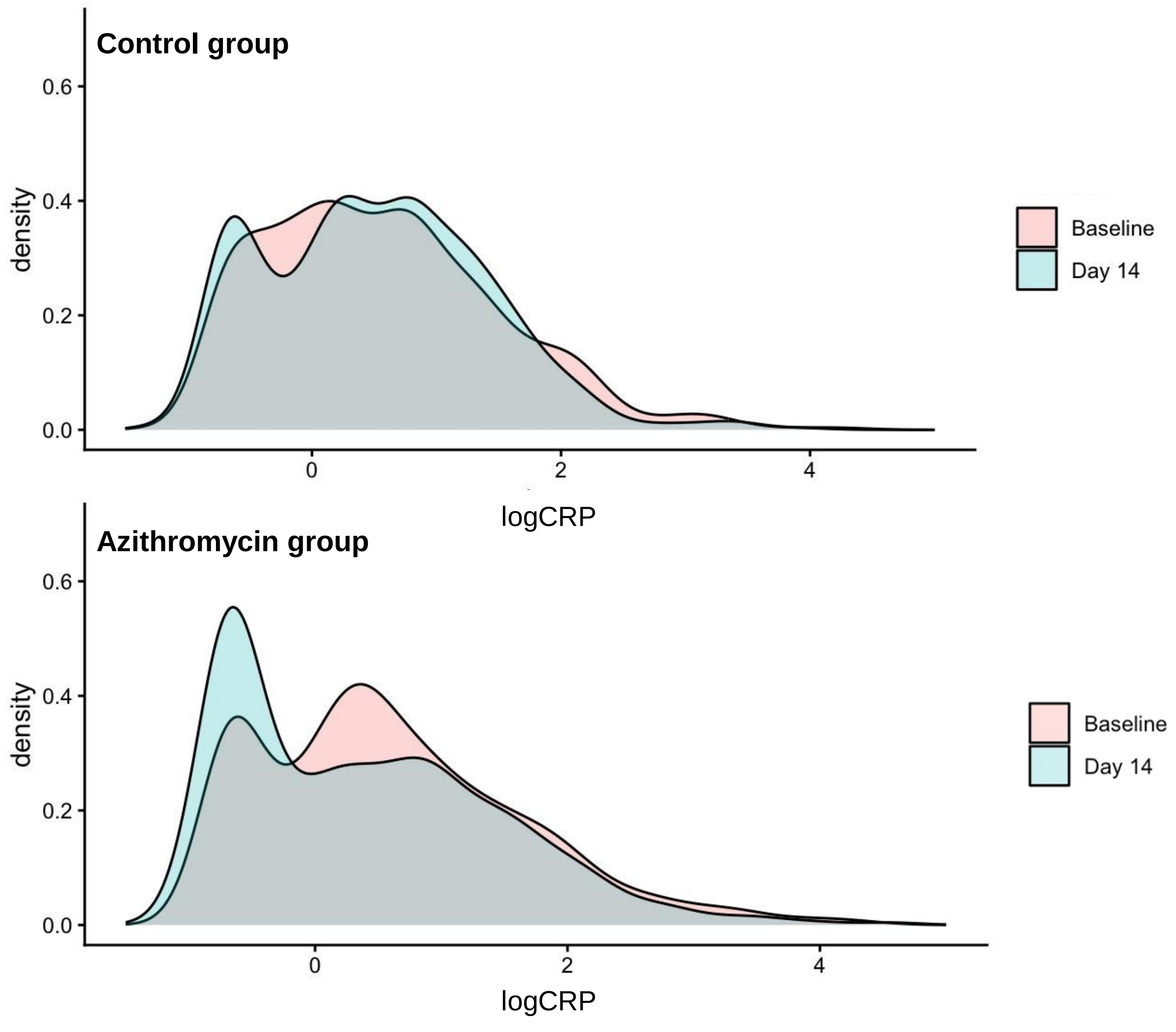

Table S1. Prevalence of elevated CRP cut-offs at day 14

**Variable**	**Control**	**Azithromycin**	**Risk ratio (95% CI)**	***P*-value**
Prevalence (%) or CRP>1 mg/L	69.5	55.6	0.81 (0.72 to 0.91)	<0.001
Prevalence (%) of CRP>5 mg/L	10.1	13.3	1.17 (0.77 to 1.77)	0.46
Prevalence (%) of CRP>10 mg/L	1.7	3.9	1.91 (0.58 to 6.32)	0.29

CRP = C-reactive protein
